# Detection of Particulate Matter of Size 2.5 μm with a Surface-Acoustic-Wave Sensor Combined with a Cyclone Separator

**DOI:** 10.3390/mi9080398

**Published:** 2018-08-12

**Authors:** Fung-Yu Kuo, Ying-Chen Lin, Ling-Yi Ke, Chuen-Jinn Tsai, Da-Jeng Yao

**Affiliations:** 1 Institute of NanoEngineering and MicroSystems, National Tsing Hua University, Hsinchu 30013, Taiwan; fency726@gmail.com (F.-Y.K.); lingyi0412@gmail.com (L.-Y.K.); 2 Institute of Environmental Engineering, National Chiao Tung University, Hsinchu 30013, Taiwan; eva770117.ev99g@nctu.edu.tw (Y.-C.L.); cjtsai@mail.nctu.edu.tw (C.-J.T.); 3 Department of Power Mechanical Engineering, National Tsing Hua University, Hsinchu 30013, Taiwan

**Keywords:** cyclone separator, surface-acoustic-wave sensor, environmental sensing, PM2.5

## Abstract

A device to monitor particulate matter of size 2.5 μm (PM2.5) that has been designed and developed includes a surface-acoustic-wave sensor operating in a shear horizontal mode (SH-SAW) combined with a cyclone separator. In our tests, aerosols generated as incense smoke were first separated and sampled inside a designed cyclone separator; the sampled PM2.5 was then introduced into the sensing area of an SH-SAW sensor for detection. The use of microcentrifuge tubes as a cyclone separator effectively decreases the size and power consumption of the device; the SAW sensor in a well design and operating at 122 MHz was fabricated with MEMS techniques. After an explanation of the design of the cyclone separator, a simulation of the efficiency and the SAW sensor detection are discussed. A microcentrifuge tube (volume 0.2 mL, inlet and outlet diameters 0.5 mm) as a separator has separation cutoff diameters 50% (*d*_50_) at 2.5 μm; the required rate of volumetric flow at the inlet is 0.125 LPM, according to simulation with computational fluid dynamics (CFD) software; the surface-acoustic-wave (SAW) sensor exhibits sensitivity approximately 9 Hz/ng; an experiment for PM2.5 detection conducted with the combined device shows a strong positive linear correlation with a commercial aerosol monitor. The limit of detection (LOD) is 11 μg/m^3^ with sample time 160 s and total detection duration about 5 min.

## 1. Introduction

The potential health effect caused by particulate matter in air, also called aerosol, is significant at present as air pollution becomes severe. Anthropogenic and biological aerosols, such as those found in haze, smoke and bacteria, fungi found in facilities for treatment of wastes and agricultural activity, can invade the human respiratory system and cause asthma, lung, and cardiovascular diseases [1,2]. In 2004, the contribution of ambient and indoor air pollution to lung cancer was estimated by World Health Organization’s Globe Burden of Disease to cause, worldwide, 62,000 and 16,000 deaths per year; airborne PM is designated a carcinogen in Group 1 by WHO International Agency of Research on Cancer [3]. In 2012, diseases contributed by air pollution caused 7 million deaths [4]; guidelines and regulation of PM have hence been established by governments and organizations worldwide. A common classification of PM is based on its size, such as PM2.5 for which the aerodynamic diameters (AD) are smaller than 2.5 μm. WHO set guideline values of PM2.5 as annual mean concentration 10 µg/m^3^ and 24-h mean concentration 25 µg/m^3^, but few countries can achieve this goal [5]. The impact of the particles collected on the surface of the acoustic sensor induces a gravimetric effect that modifies the conditions for acoustic-wave propagation [6]. The development of a device that is convenient, cheap, and suitable for monitoring the personal exposure to aerosols in real time thus becomes an important task [7].

PM2.5 monitoring is currently generally conducted using instruments based on, for example, beta attenuation or a tapered-element oscillating microbalance, which are expensive, time-consuming, and bulky, as shown in Table 1. Some miniature PM2.5 sensor modules for personal use are available on the market; most are based on the principles of light scattering and piezoelectricity. For light scattering, such a detection mechanism is in accordance with an assumption of the physical parameters and size distribution parameters of the aerosol, which causes an uncertainty of measurement because of the natural variability of PM2.5 and leads to a poor accuracy of a sensor [8,9]. Based on the sensing mechanism of a mass-loading effect, a device with a surface-acoustic-wave sensor operating in a shear horizontal mode (SH-SAW) can effectively decrease this inaccuracy and exhibit a greater sensitivity than another piezoelectric sensor such as a quartz-crystal microbalance (QCM). A surface-acoustic-wave (SAW) device can be fabricated in a simple process and operated without a complicated setup or expensive instruments [10,11].

In additional to the sensitivity and reliability of the sensor itself, without an effective aerosol sampler most monitors might have a large disturbance signal from aerosols of kinds other than PM2.5. In this work, a microcentrifuge tube was developed into a replaceable cyclone separator for PM2.5 sampling. For a comparison with the required flow rate of commercial separators, for a commercial separator to achieve a cutoff diameter at 2.5 μm the flow rate is about 2–4 LPM [12]; our separator with a much smaller required flow rate is compatible with a micro pump that can decrease the size of a complete device.

## 2. Materials and Methods

### 2.1. Fabrication of SH-SAW Chips

**Figure 1:**
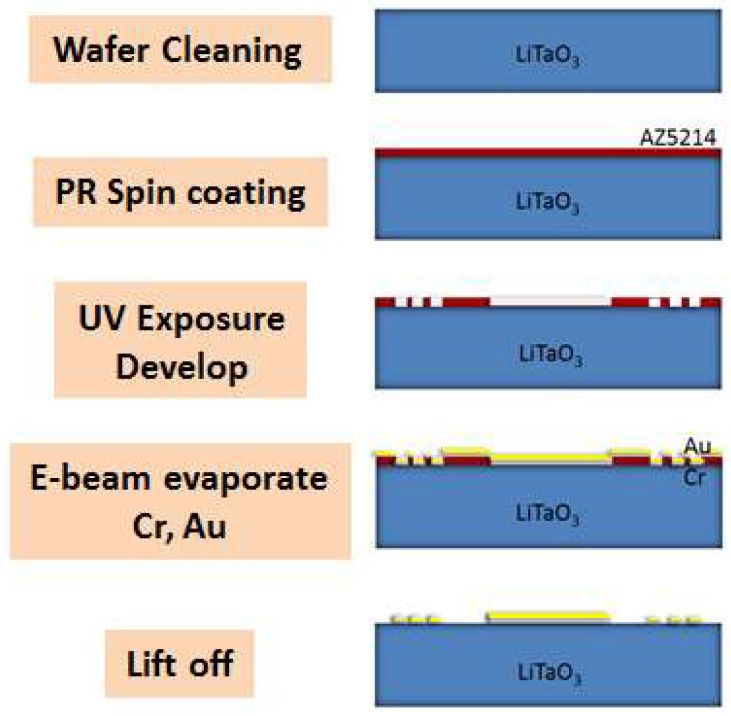
Fabrication of SH-SAW chips.

The SH-SAW chip was designed with central frequency 122.4 MHz. A lithium-tantalate (LiTaO_3_) piezoelectric substrate with a large electromechanical coupling coefficient (*K*^2^) was chosen [13]; the interdigital transducer (IDT) made from gold was patterned with photolithography and an e-beam evaporator. The IDT consist of 50 pairs with finger width 8.5 μm, and deposited thickness 100 nm; an overall die size is 13.4 mm × 7.4 mm. The process included three main steps: (a) photolithography, (b) e-beam evaporation and (c) lift-off, as shown in Figure 1.

(a) Photolithography

The LiTaO_3_ wafer was first cleaned according to the following standards. The wafer was given a positive photoresist (AZ5214, MicroChemicals GmbH, Ulm, Germany) at 3000 rpm for 30 s. The wafer was then baked at precisely 100 °C for 1 min and exposed to ultraviolet (UV) light. The pattern was developed and inspected with a microscope to verify the completeness of the IDT structure.

(b) E-beam evaporation

After the photolithographic process, Cr (thickness 20 nm) was deposited first on the substrate as an adhesive layer; Au (thickness 100 nm) was deposited second as a major structure with e-beam evaporation.

(c) Lift-off

After the evaporation, the wafer was immersed and sonicated in acetone to remove unnecessary photoresist, and the pattern was defined. The finished wafer was diced into chips with laser cutting.

### 2.2. Cyclone Separator Design and Simulation

**Figure 2:**
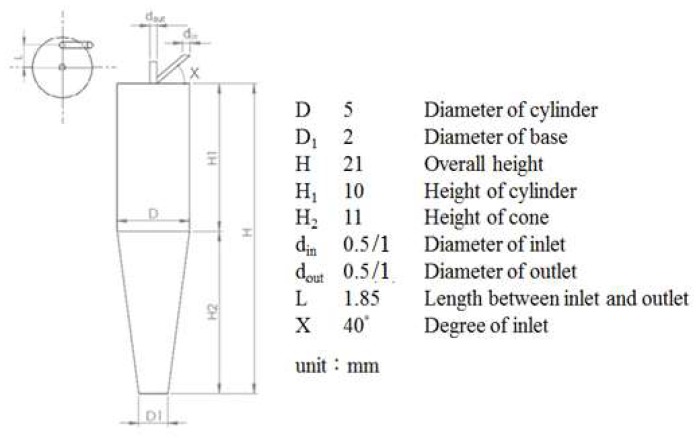
Dimensions and design parameters of microcentrifuge tube (0.2 mL) as a cyclone separator.

Lindsley and coworkers were the first to use a microcentrifuge tube as a replaceable cyclone separator for bioaerosol sampling [14]. Based on this research, we developed a microcentrifuge tube (volume 0.2 mL) into a smaller PM2.5 cyclone separator [15,16]. In our work, aerosols are first introduced into the tube from the slanted inlet and separated via a vortex; particles of average diameter (AD) greater than 2.5 μm are collected in the bottom of the tube, whereas particles with AD smaller than 2.5 μm are transported to the upper outlet and can be detected with the sensor. The dimensions of the tube are fixed; two parameters of the inlet and outlet sizes are designed such that the ratio between tube, inlet and outlet is about *D*: *d*_in_/*d*_out_ = (A) 10:1 and (B) 5:1, as shown in Figure 2. 

**Figure 3:**
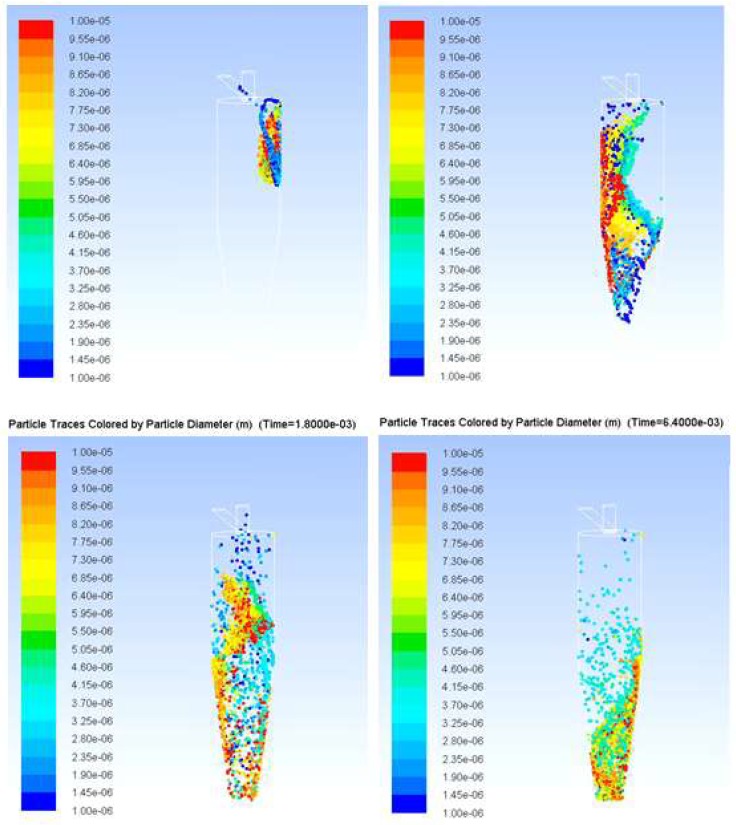
Particle traces colored according to the diameter at varied flow times. The simulation photos have shown the different size particle in the cyclone.

The optimum operating flow velocity of a cyclone separator is between 10 and 20 m/s; for operation at a small flow rate, the inlet and outlet diameters are decreased, although the separation efficiency might also decline. The designed cyclone was hence simulated with software for computational fluid dynamics (CFD) to find the best balance between efficiency and operational consumption. CFD software (Fluent, ANSYS Fluent Inc., Lebanon, NH, USA) with the finite-element method was used. The simulation had three major parts. (1) The cyclone geometry was drawn using 3D CAD software (SolidWorks) and meshed in pre-processing; the number of mesh elements was about a million. (2) Numerous solution parameters were set, including phase models. The air was set at normal temperature and pressure as a continuous phase using a Reynolds stress model to solve with inert particles (0.1–6 μm) as a discrete phase using a model of discrete particulate matter (DPM) to solve; an uncoupled calculation was chosen for them because the volume fraction of particles in air is small, ≤12%, in which case the continuous phase is not impacted by the presence of the discrete phase. For the phase characteristics, the discrete phase density is the average bulk density of the PM2.5 [17,18]. Previous PM2.5 studies reported particle densities mainly between 1 g/cm^3^ and 3 g/cm^3^ [19,20]. For the boundary conditions, particles became trapped when they reached the surface of the outlet. (3) After the calculation, an analysis of the flow field and particle trajectories analysis was undertaken in post-processing, as shown in Figure 3. The particles were separated with the vortex as a function of time. The particles of average diameter (AD) greater than 2.5 μm were collected at the bottom of the tube, whereas particles with AD smaller than 2.5 μm were transported to the upper outlet. The number of suspended particles was hence equal to the number of meshes of the face; the particles that were calculated to reach the boundary could be trapped or could escape. The position of the particles in the separator varied with time depending on the size. The particles entered the separator at the maximum intake velocity; the reverse flow generated when reached the bottom brought together the particles of small diameters; the particles of large diameter were deposited below. The particle cutoff diameters (*d*_50_) were near the experimentally determined values. The number of particles separated in the outlet is plotted as a cumulative-volume distribution of particle size so that the *d*_50_ could be compared [21].

### 2.3. Sensing System

**Figure 4:**
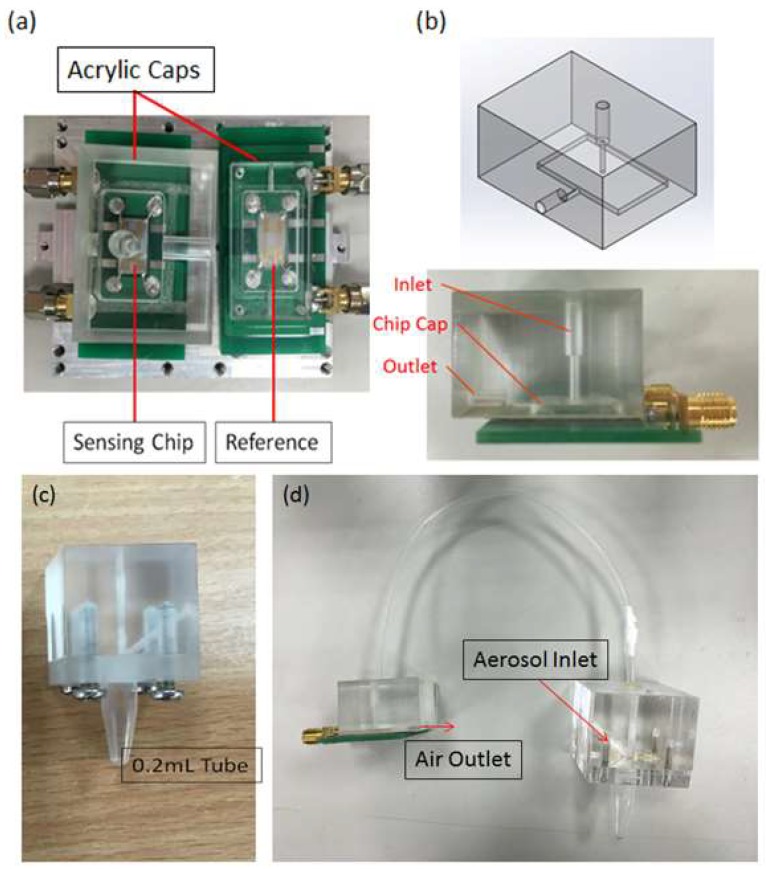
(**a**) Two-port SH-SAW sensors. (**b**) Sensing chip cap. (**c**) Assembled cyclone separator using a microcentrifuge tube (0.2 mL) and channels made of acrylic. (**d**) PM2.5 detection device with a SH-SAW sensor combined with a cyclone separator.

**Figure 5:**
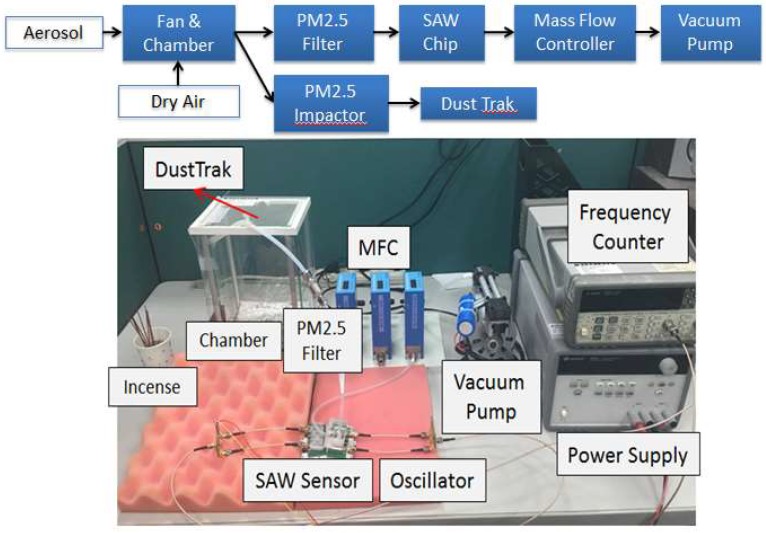
Experimental setup.

The SH-SAW sensor was first tested to identify its sensitivity to PM2.5. Two-port sensors were used in the experiment, one of which is considered a reference to monitor and to compensate for the variable ambient condition, and the other is for PM2.5 detection, as shown in Figure 4a. The setup of the detection experiment is shown in Figure 5; an aerosol was generated on burning incense sticks, which renders a distribution of particle size from 0.06 to 2.5 μm, with average bulk density 1.1 g/cm^3^ [22]. Incense smoke was exhausted with a fan into a glass chamber (20 × 15 × 15 cm^3^), with dry air supplied to adjust its concentration from 10 to 200 μg/m^3^. The chamber connected into two pipe channels, of which one led directly to the SH-SAW sensor with a PM2.5 filter before the sensor and an acrylic cap covering the chip to ensure that the particles can strike and adhere to the sensing zone before leaving to the outlet, as shown in Figure 4b; the flow was controlled with a mass-flow controller (MFC) and a vacuum pump; the other channel was connected into a commercial monitor (DustTrak Aerosol Monitor 8530, TSI Inc., Shoreview, MN, USA) to provide a reference of real-time PM2.5 mass concentrations at every second. The sensors were connected to an oscillator circuit, a power supply, and a frequency counter; a computer recorded the frequency signal every 2 s.

After identifying the sensitivity of the SH-SAW sensor, we tested the combined device of the SH-SAW sensor and cyclone separator (0.2 mL), as shown in Figure 4c,d; the upper outlet of the separator was connected to the inlet of a chip cap, for separated particles to flow to the sensor. A cyclone separator (0.2 mL) was used as a substitute for a PM2.5 filter; the experiments were conducted with and without a cyclone separator to verify its efficiency. The experimental parameters of the separator depended on the results of the CFD simulation.

## 3. Results

### 3.1. Simulation of the Efficiency of the Cyclone Separation

**Figure 6:**
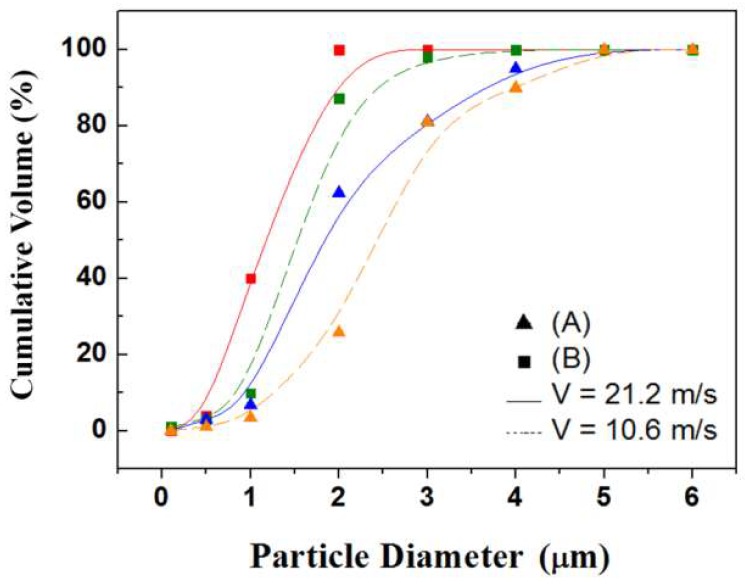
Comparison of separation efficiency of the tube (0.2 mL). The results indicate that design (B) generally had a separation efficiency better than that of design (A).

Two parameters of inlet and outlet sizes were designed such that the ratio of the tubes was about (A) 10:1 and (B) 5:1 and were simulated under operating flow rates 10.6 m/s and 21.2 m/s separately. Figure 6 shows a comparison of the separation efficiency of the microcentrifuge tube (0.2 mL). With inlet flow velocities 21.2 and 10.6 m/s, the tube with design (A) had *d*_50_ at 1.8 μm and 2.5 μm, respectively; the tube with design (B) had *d*_50_ at 1.1 μm and 1.5 μm, respectively. From the simulation results we concluded that tubes with design B have a separation efficiency better than that of design A, but the tube with design A still has *d*_50_ at PM2.5 at inlet flow velocity 10.6 m/s; the required volumetric flow rate is 0.125 LPM. It is hence impractical to decrease the size of the inlet and outlet ports without limit to decrease the flow demand, but the 0.2-mL centrifuge tube was designed (A) to achieve *d*_50_ equal to PM2.5 at inlet flow rate 10.6 m/s; the required flow rate was only 0.125 LPM.

### 3.2. SH-SAW Sensor Aerosol Experiment

#### 3.2.1. Test of Sensor Sensitivity 

**Figure 7:**
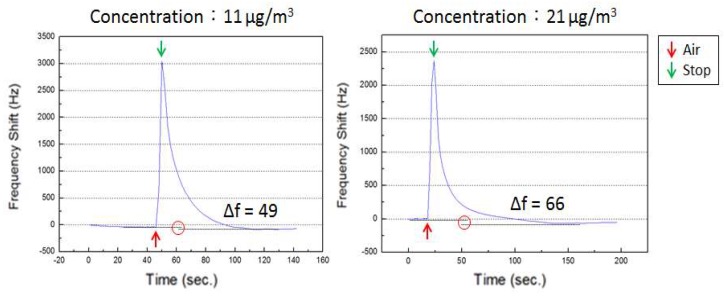
Frequency response curve of the SH-SAW sensor to incense smoke at varied concentration.

At the initial ventilation, the frequency increased greatly, because of the mass-loading effect caused by the air flow, but, when the air flow stopped, the frequency slowly returned to the original value because of the non-ventilation condition. At the same time, the suspended particles became slowly deposited on the surface of the SAW, which on returning to the original value continued to decrease. The SH-SAW sensor can detect a mass-loading of particles that deposit on the sensing zone and transmit a frequency signal; the frequency data are processed including compensating the reference value, and zero as the initial frequency value. As shown in Figure 7, the moment that the aerosol and air flow into the sensor causes a large frequency change, but the sensor returns to a stable state when the air stops; the deposited particles leave a frequency shift, with a response interval about 1–2 min. On comparison of the frequency shift with the mass concentration measured with the aerosol monitor, the sensitivity (*S*) of the SH-SAW sensor was calculated with Equation (1), in which Δ*f* is the frequency shift (Hz), *mc* is mass concentration (μg/m^3^), *Q* is the input flow rate (m^3^/s), and *t* is the sample interval (s).

(1) $$S=\frac{\Delta f}{mc\times Q\times t}$$

**Figure 8:**
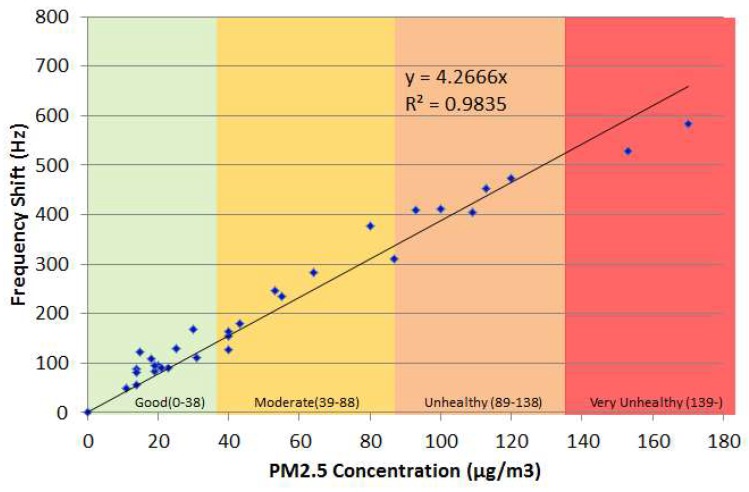
Frequency response of SH-SAW sensor to varied PM2.5 concentration.

Figure 8 shows the frequency shift responses of an SH-SAW sensor to incense smoke versus mass concentrations measured with a commercial detector (TSI DustTrak, Shoreview, MN, USA); the tested mass concentration is from 11 to 170 μg/m^3^; the limit of detection is 11 μg/m^3^ at frequency shift response 49 Hz. We focused on an experiment with a small concentration (10-50 μg/m^3^) that corresponds to a PM2.5 concentration in an actual environment when the air is in effective circulation with no obvious PM source such as cigarette or incense smoke. The coefficient of determination is calculated to be 0.9835; converting the frequency to the mass sensitivity of the sensor is approximately 9 Hz/ng with flow rate 3 LPM and sampling interval 10 s.

#### 3.2.2. Test of an SH-SAW Sensor Combined with a Cyclone Separator 

After identifying the PM2.5 mass sensitivity of the SH-SAW, we determined the mass concentration of the detected PM2.5 on measuring the frequency shift; the mass concentration was calculated with Equation (1). 

The sensitivity was 9 Hz/ng; other experimental parameters are shown in Table 2. The cyclone separator (0.2 mL, with inlet and outlet diameters 0.5 mm), at operating flow rate 0.125 LPM with *d*_50_ at 2.5µm was chosen to replace the PM2.5 filter for the test. Because the operating flow rate was small, the sample interval was increased to 160 s for sufficient particles to deposit. The test without a cyclone and other PM2.5 separator was conducted with operating flow rate 3 LPM and sampling interval 10 s, to verify the necessity and efficiency of the cyclone separator (0.2 mL).

**Figure 9:**
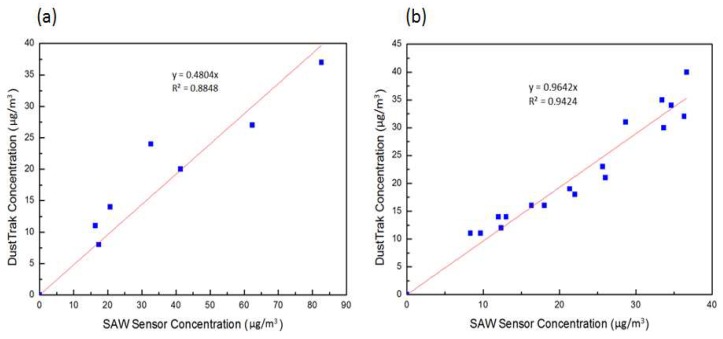
(**a**) Aerosol test of an SH-SAW sensor with no PM2.5 separator. (**b**) Aerosol test of SH-SAW sensor with a cyclone separator (0.2 mL).

Figure 9a shows the mass concentration detected with the SH-SAW sensor versus the mass concentration measured with the reference (DustTrak); the error of the SH-SAW sensor measurement was much greater than that of the aerosol monitor when the sensor was tested without a PM2.5 separator, because particles larger than 2.5 μm also deposited in the sensing zone and influenced the frequency change. An error of this kind is difficult to calibrate with a calculation or other method because of the variability of aerosols depending on the source, an environment with varied composition and its size distributions; for this reason, an appropriate PM2.5 separator is necessary. 

Figure 9b shows the mass concentration detected with an SH-SAW sensor and a cyclone separator (0.2 mL) versus mass concentration measured with the reference (DustTrak); the operating flow rate was 0.125 LPM, the sampling interval was 160 s. The coefficient of determination was calculated to be 0.9642. A strong positive linear correlation between the SAW sensor and the reference (DustTrak) shows that the cyclone separator (0.2 mL) effectively excluded the influence of particles other than PM2.5. The required flow rate of this design was decreased but the total detection period was increased to 4–5 min; the ability of real-time sensing was hence decreased.

## 4. Conclusions

In this work, we developed a PM2.5 monitoring device. The shear horizontal- mode surface-acoustic-wave sensor fabricated with a MEMS technique exhibited sensitivity 9 Hz/ng to PM2.5; the limit of detection was 11 μg/m^3^. A cyclone separator with a microcentrifuge tube (volume 0.2 mL, diameter 0.5 mm of inlet and outlet) had a separation cutoff diameter at 2.5 μm; the required flow rate was 0.125 LPM. The power consumption and size (volume) were much decreased relative to an existing PM2.5 separator. The aerosol experimental data measured with the combined device show a strong positive linear correlation with a commercial aerosol monitor, with sample interval 160 s, total detection period 5 min. The results show a great potential for a real-time PM2.5-monitoring device for the environment.

For a further pratical application, the design and fabrication of the SH-SAW sensor will be improved on decreasing the IDT width to achieve a desired wave penetration depth (~2.5 μm), so that the PM2.5 sensitivity and ability of the sensor will improve for real-time sensing [23].

## Figures and Tables

**Table 1:** Comparison of aerosol monitoring instruments.

Principle	Instrument	Resolution (m/m^3^)	Advantages	Volume/Mass	Price (NTD)
Optical	TSI DustTrak 8532	1	Real-time measurement (1 s); Portable	6 × 16 × 10cm^3^/1 kg	$300,000
Beta Attenuation	Met-One BAM 1020	0.1	Continuous data; Error-handling software	31 × 43 × 40cm^3^/24.5 kg	$580,000
Oscillating Microbalance	Thermo RP 1400	0.1	Simple design principle	28 × 22 × 43cm^3^/20 kg	$550,000
Spectrometer	GRIMM 180	0.1	31 size channels	27 × 36 × 48cm^3^/18 kg	$850,000

**Table 2:** Parameters for the cyclone separator test.

Cyclone Size	*d*_in_/*d*_out_/mm	Operating Flow Rate/LPM	*d*_50_/µm	Sample Time/s
Without Cyclone	-	3	-	10
0.2 mL	0.5/0.5	0.125	2.5	160
